# Sex-specific genetic variances in life-history and morphological traits of the seed beetle *Callosobruchus maculatus*

**DOI:** 10.1002/ece3.56

**Published:** 2012-01

**Authors:** Lára R Hallsson, Mats Björklund

**Affiliations:** Animal Ecology/Department of Ecology and Genetics, Evolutionary Biology Centre (EBC)Uppsala University, Uppsala, Sweden

**Keywords:** Additive genetic variance, breeding design, genetic correlation, sexual dimorphism, sex-specific genetic effect

## Abstract

Knowledge of heritability and genetic correlations are of central importance in the study of adaptive trait evolution and genetic constraints. We use a paternal half-sib-full-sib breeding design to investigate the genetic architecture of three life-history and morphological traits in the seed beetle, *Callosobruchus maculatus*. Heritability was significant for all traits under observation and genetic correlations between traits (*r*_A_) were low. Interestingly, we found substantial sex-specific genetic effects and low genetic correlations between sexes (*r*_MF_) in traits that are only moderately (weight at emergence) to slightly (longevity) sexually dimorphic. Furthermore, we found an increased sire (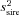) compared to dam (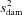) variance component within trait and sex. Our results highlight that the genetic architecture even of the same trait should not be assumed to be the same for males and females. Furthermore, it raises the issue of the presence of unnoticed environmental effects that may inflate estimates of heritability. Overall, our study stresses the fact that estimates of quantitative genetic parameters are not only population, time, environment, but also sex specific. Thus, extrapolation between sexes and studies should be treated with caution.

## Introduction

In order to understand the evolutionary response of a population, knowledge of the additive genetic variance (*V*_A_) or heritability (*h*^2^; which in its narrow sense is the ratio of additive genetic and phenotypic variance, Falconer and Mackay 1996) is crucial, since *V*_A_ determines the rate of evolutionary change, given the selection acting on the population (Falconer and Mackay 1996). In addition, genetic correlations are of central importance in the study of adaptive trait evolution. Genetic correlations can facilitate or impede the response selection, because characters evolve in response to selection as a direct consequence of the forces of selection operating on the characters themselves and as an indirect consequence of selection operating on all genetically correlated traits (Lande and Arnold 1983; Falconer and Mackay 1996; Lynch and Walsh 1998). Genetic correlations can be measured between traits, but also between sexes for homologous traits. Strong genetic correlations (of one or close to one) between traits/sexes can potentially constrain independent trait evolution/the evolution of sexual dimorphism (Lande 1980; Falconer and Mackay 1996; Reeve and Fairbairn 1996; Fairbairn 1997). A genetic correlation of less than one suggests partial independence of traits and the possibility for independent evolution of trait/sexes (Lande 1980, 1987; Falconer and Mackay 1996; Reeve and Fairbairn 2001). Thus, estimating *V*_A_ and/or *h*^2^ and genetic correlations has been a main focus in quantitative genetic studies (Lynch and Walsh 1998).

Different experimental designs have been developed and used to estimate *h*^2^ in natural, seminatural, and laboratory populations, such as parent–offspring regression, full-sib, and half-sib analysis, and the “animal model” (pedigree-based approaches) has been increasingly used to estimate *h*^2^ in natural populations (Kruuk 2004; Kruuk et al. 2008). Estimates can be biased due to environmental, nonadditive genetic, and parental effects. The aim of a breeding design is to eliminate or estimate these effects. A common design used in this respect is a nested paternal half-sib design (Falconer and Mackay 1996), where a set of males is mated to several females each, creating full-sibs from each female and half-sibs between females mated with the same male. The variance among half-sib families is used to estimate additive genetic variance in the population, as the sire variance component is assumed to be an unbiased estimator of the additive genetic variance. In that way, maternal effects (offspring has different mothers but the same father) and common environmental effects (offspring is randomized in the environment they are reared in) are eliminated, and possible paternal effects are assumed to be absent. However, this design has the potential drawback that if there are paternal effects (Hunt and Simmons 1998, 2000), these can inflate the estimate of additive genetic variance, that is, the offspring measured (half-sibs) have the same father.

In this study, we use a paternal half-sib-full-sib design to investigate the genetic architecture of weight at emergence, developmental time, and longevity in the seed beetle *Callosobruchus maculatus* (Coleoptera, Bruchidae), examining sexes separately. Several previous studies (Fox 1993a, 1998; Fox et al. 2004) have successfully used this design and shown significant heritability for the same and similar traits in *C. maculatus* and related species. However, as apparent from the results of our study, there are substantial sex-specific genetic effects on life-history and morphological traits and low genetic correlations between traits and sexes, which contradict findings of previous studies. Moreover, we found suggestive evidence that an environmental effect is present in the estimate of the sire variance component. The surprising results highlight that sexes should not be assumed to be the same, stresses the fact that estimates of quantitative genetic parameters are population, environment, time and sex specific, and raises the issue of the presence of unnoticed environmental effects in estimates of heritability.

## Material and Methods

We used the seed beetle C. maculatus, which is a cosmopolitan pest of stored legumes (Fabaceae). Mated female seed beetles cement their eggs to the surface of host bean (Messina 1993) and newly hatched larvae burrow into the seed. The larval development and pupation are completed entirely within a single host seed. Adults emerging from the bean are well adapted to storage conditions, requiring neither food nor water to reproduce. They live for an average of 10 days (without food or water supply); their entire life cycle from egg to egg is completed in 21–24 days at 30°C (Fricke 2006). We used a mixed strain (Nigerian Mix) of *C. maculatus*. The Nigerian mixed strain was established in our laboratory at Uppsala University in 2002 by mixing three beetle populations. We received the three beetle populations from Dr. Peter Credland (University of London). Populations had been collected in large numbers from three adjacent locations in Nigeria (Oyo, Zaira, and Lossa), Africa and had been kept in the laboratory prior to their transfer to our laboratory for 2 years (approximately 24–30 generations). Beetles from the Nigerian mixed strain were kept on black-eyed cowpeas (*Vigna unguiculata*) as a host with 250–350 randomly chosen adult beetles transferred to 120–140 g of host medium every new generation in incubators under constant conditions at 30°C and 45% (±10%) relative humidity.

### Breeding design

In order to estimate heritability and sire/dam variance components for developmental time and longevity (life-history traits) and weight at emergence (morphological trait), we conducted a paternal half-sib breeding design (Falconer and Mackay 1996). A random sample of 50 males (sires) was mated to a unique set of five randomly chosen females (dams), resulting in 250 families. The offspring from each dam were raised and the phenotypic traits of interest measured. The variance among half-sib families represents one-fourth of the additive genetic variance (*V*_A_) and can therefore be used to estimate V_A_ and the narrow-sense heritability *h*^2^ (Lynch and Walsh 1998). Virgin males and females were collected from isolated host beans of the basic population and one male was presented to five females on a 92 × 16 mm petri dish for 24 h. After 24 h, the male was removed and females placed in a 92 × 16 mm petri dish filled with 25 g (∼100 beans) each. Females were allowed to lay eggs for 24 h before they were removed. The high number of beans resulted in females laying approximately 1–2 eggs per bean, thus within bean larva competition is minimized. Beans with eggs were split into two groups: beans with attached eggs of each female were divided into two virgin chambers (one chamber per group, 25 beans in each chamber). The first group was used to collect data for developmental time and weight at emergence. Adults emerging were collected at three sampling events (corresponding to early, median, and late emerging individuals). One sample was taken every second day during the emergence period of 7 days. Sampling started when 10 individuals had emerged. Emerging individuals were sexed, collected in Eppendorf tubes (males and females separately), counted and stored in a freezer. As a measure of individual weight, dry weight was recorded. In *C. maculates*, dry body weight has been shown to be highly correlated with wet body weight for both female and male (Guntrip et al. 1997). Individuals were dried in an oven at 50°C for 2 days and their weight obtained to the nearest 0.01 mg (using a Sartorius Genius Microbalance model ME235P - OCE, Sartorius AG, Göttingen, Germany). The second group was formed to collect longevity data. Sampling began when the first individuals started dying (8 days after the preceding emergence peak). Mortality was checked every second day until all individuals were dead; this resulted in 12 sampling events over a period of 23 days.

In total 5732 offspring (3138 males and 2594 females) were reared. Developmental time and weight at emergence data (Group 1) were recorded from 2908 offspring (1643 males and 1265 females), reared from 165 full-sib families (45 sires and 165 dams). Longevity data (Group 2) were collected from 2824 offspring (1495 males and 1329 females), reared from 203 full-sib families (47 sires and 203 dams).

### Statistical analyses

All statistical analysis was conducted in R version 2.13.0 (R Development Core Team 2011). We fitted a linear mixed model (using the lme4 package in R; Bates and Maechler 2009) to the data, including sex as fixed and sire and dam as random factors; dam is nested within sire due to the breeding design. The effect of sex was estimated by a likelihood ratio test (LRT). We estimated variance components and residual variances for both sexes and males and females separately using restricted maximum likelihood (REML) procedure. REML was chosen over an analysis of variance (ANOVA) analysis because there was a slight unbalance in the data (due to the fact that there were unequal numbers of dams mated to each sire and there were unequal numbers of offspring per dam) and REML analysis is known to be less sensitive to unbalanced data (Lynch and Walsh 1998). REML estimates produce the observational variance components (i.e., variance components due to different factors in the mating design [e.g., sire, dam]) directly, in contrast to ANOVA estimates, where the observational (sire, dam) variance components have to be calculated from mean squares first (Lynch and Walsh 1998). Confidence intervals for sire (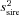) and dam (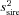) variance component were accessed using the profile function in a developmental version of the lme4 package (lme4a, http://r-forge.r-project.org/). The significance of variance components was assessed using LRTs.

In the half-sib design typically the sire variance component is used to estimate heritability. The sire variance component estimates the phenotypic covariance of half sibs. It includes only additive genetic variance (*V*_A_) and is unbiased by dominance, maternal, and environmental (offspring randomized across the environment) effects (Equation 1; Falconer and Mackay 1996). The dam variance component estimates the phenotypic covariance of full sibs minus the phenotypic covariance of half sibs. It typically includes *V*_A_, but also dominance (*V*_D_) and maternal or other environmental effects (*V*_Ec_) (Equation 2; Falconer and Mackay 1996). Analogously to the *V*_A_ estimate from the 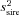, *V*_A_ can be estimated as four times the 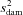. However, the estimate is more likely to be confounded by nonadditive effects. Both sire and dam variance includes epistatic variance, but these effects are small, especially in the case of the sire variance and are therefore assumed to be absent.


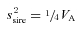
(1)


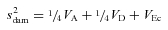
(2)

We calculated heritability based on sire and dam variance components by dividing four times the sire or dam variance by the phenotypic variance (including sire, dam, and residual variances). The least biased estimated of heritability in this breeding design is based on the sire variance component (Falconer and Mackay 1996; Lynch and Walsh 1998). Therefore, we continue our interpretation and discussion of results based on this estimate and will refer to it as heritability (*h*^2^) from hereon. Confidence intervals of the heritability estimates were assessed using parametric bootstrapping. We conducted a randomization test in order to investigate sex differences in variance components (Good 2005); first, we calculated the observed difference between males and females variance components as our test statistic, we then randomized sex, refitted the model, and recalculated the difference. In this way, we get an empirical distribution of sex differences in variance components under the null hypothesis of no sex difference. The proportion of permutations (in the null model; sex random) that gives sex differences equal or more extreme than the observed difference is the *P*-value for sex effects on variance components (two-tailed test).

We compared sire and dam variance components for each trait and sex by evaluating their confidence intervals. This gives an estimate of eventual presence of nonadditive genetic and environmental effects in either variance component. A dam variance component that is significantly larger than the sire variance component indicates the presence of nonadditive effects (dominance, epistatic maternal, and other environmental effects) (e.g., Bubliy and Loeschcke 2002). Analogously, a sire variance component that is significantly larger than the dam variance component suggests the presence of paternal nonadditive and/or environmental effects. This interpretation assumes that family environment effects have no effect on within full-sibship variation or cause similarity between family members. A family environment effect can potentially increase the within full-sibship variation though (e.g., sibling competition). However, we can exclude an effect of, for example, sibling competition, since within bean larva competition was minimized in the breeding design. To test for the concordance of results between sampling periods, we used the Friedman test that is a nonparametric repeated ANOVA.

In order to calculate genetic correlations between sexes and traits, we fitted three two-trait linear mixed random slope models (i.e., a model for each two trait combination) to the data using a Bayesian approach (MCMCglmm package, Hadfield 2010). Sex and trait identity were fitted as fixed effect predictors, and sire as well as dam (nested within sire) were included as random effects. Since we were interested in the covariances between sexes and between traits, we also fitted the interactions of sex and traits with the two random effects. For the sire identity and the dam identity random effects, we estimated unstructured variance–covariance matrices, that is one variance for each trait and sex (four variances) and all covariances between sexes and traits (six covariances), we fitted residual variances heterogeneous. We used flat priors for the fixed effects and uninformative priors for the random effects and allowed the Markov chain a burn-in period of 7000 iteration, after which we ran 60,000 iterations and sampled every 30th iteration from the posterior distribution. We calculated genetic correlations based on the posterior variance covariance matrices. The genetic correlation between the sexes (*r*_MF_) for each trait was assessed by dividing the covariance between sexes by the square root of the product of variance for each sex; we calculated the genetic correlation between traits (*r*_A_) for each sex separately by dividing the covariance between traits by the square root of the product of variance for each trait. Significance of estimates of the genetic correlation was assessed by their 95% highest posterior density (HPD) interval not overlapping zero or one, respectively.

## Results

**Figure 1:**
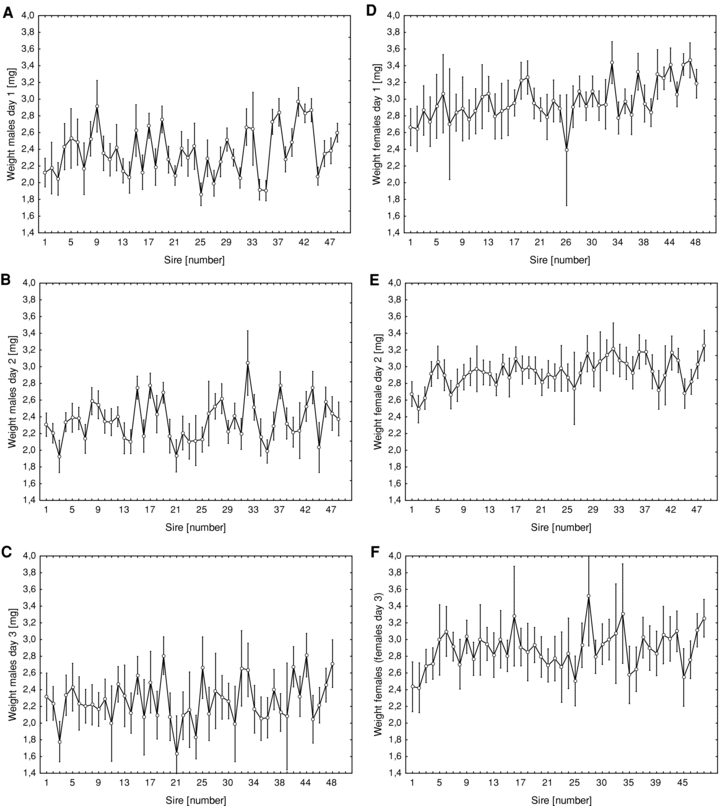
Variance in offspring weight at different days of emergence for each sire. Day 1 to day 3 represent early, intermediate, and late emerging individuals, respectively. (A) Males day 1, (B) Males day 2, (C) Males day 3, (D) Females day 1, (E) Females day 2, (F) Females day 3.

There was a significant sex effect on weight at emergence (LRT; χ^2^ = 1546.7, df = 1; *P* < 2.2 × 10^–16^), developmental time (LRT; χ^2^ = 9.65, df = 1; *P* = 0.0019), and longevity (LRT; χ^2^ = 641.84, df = 1; *P* < 2.2 × 10^–16^) with males being 27% smaller, hatching approximately 3.5 h earlier and dying 9% earlier than females. Sire and dam effects were significant for weight at emergence, developmental time, and longevity (Table 1), with sire explaining 18% (weight), 5% (developmental time), and 22% (longevity) of the variation in the phenotype. We found significant heritability for all traits measured (weight at emergence *h*^2^ = 0.75, developmental time *h*^2^ = 0.23, and longevity *h*^2^ = 0.90; for sex-specific heritability estimates and confidence intervals see Table 3). The genetic correlations between sexes (*r*_MF_) for each trait and between traits (*r*_A_) for each sex were generally low and differed significantly from one; only the *r*_MF_ for developmental time was significantly different from zero (Table 4A and B). We found sexes to be significantly different in their variance components and heritability for weight at emergence and longevity, that is there were sex-specific effect on variance components for these traits. For weight at emergence, the sire variance component was significantly greater in males compared to females (Randomization test *P* < 0.01; Table 2) and the dam variance component was significantly greater in females compared to males (Randomization test *P* = 0.016; Table 3). For longevity, the sire variance component was significantly greater in females compared to males (Randomization test *P* < 0.01; Table 2) and the residual variation was significantly higher in males (Randomization test *P* < 0.01; Table 2). Sexes did not differ in their variance components for developmental time (Table 2). Sex differences in sire and dam variance components resulted in sex differences in heritability estimates. Males had higher heritability for weight at emergence, whereas females had higher heritability for longevity (Table 3), reflecting the sex difference in sire variance components for the traits (Table 2). Comparison of sire and dam variance components within each trait and sex revealed that the sire variance component was significantly larger than the dam variance component for weight at emergence for males and for longevity for females (Table 3). There was no significant concordance of male weight at emergence at the three times of emergence (χ^2^ = 3.43, *N* = 46 families, *P* = 0.18, Friedman test, Fig. 1 A–C), but in females it was significant (χ^2^ = 8.32, *N* = 44 families, *P* = 0.016, Friedman test, Fig. 1 D–F).

**Table 1:** REML estimates of variance components; presented in percent of total phenotypic variance and as raw values for weight at emergence, developmental time, and longevity. Significance of variance components was tested using likelihood ratio tests (LRT)

			Likelihood ratio test (LRT)		
			
	Variance components (percentage)	Variance components	χ^2^	df	*P*
Weight at emergence^1^					
Sire	18.7	0.004	659.35	1	<2.2 × 10^–16^
Dam	8.9	0.002	125.59	1	<2.2 × 10^–16^
Residual	72.3	0.018			
Developmental time					
Sire	5.6	0.114	192.65	1	<2.2 × 10^–16^
Dam	7.6	0.153	58.78	1	1.76 × 10^–16^
Residual	86.6	1.742			
Longevity^2^					
Sire	22.5	0.006	727.69	1	<2.2 × 10^–16^
Dam	8.0	0.002	80.05	1	<2.2 × 10^–16^
Residual	69.4	0.019			

1 Weight at emergence data are log transformed.

2 Longevity data are fifth square-root transformed.

**Table 2:** REML estimates of sex-specific variance components; presented in percent of total phenotypic variance and as raw values for weight at emergence, developmental time, and longevity. Sex differences in variance components were tested using randomization tests

	Variance components (percentage)		Variance components		
			
	Males	Females	Males	Females	Randomization test (sex difference) *P*-value
Weight at emergence^1^					
Sire	28.0	14.5	0.009	0.002	<0.01
Dam	8.4	15.0	0.002	0.002	0.016
Residual	63.4	70.4	0.020	0.010	0.03
Developmental time					
Sire	4.1	6.2	0.083	0.122	0.34
Dam	8.1	8.4	0.167	0.167	0.92
Residual	87.7	85.3	1.797	1.686	0.43
Longevity^2^					
Sire	17.3	35.6	0.004	0.011	<0.01
Dam	9.1	5.5	0.002	0.001	0.25
Residual	73.4	58.7	0.018	0.018	<0.01

1 Weight at emergence data are log transformed.

2 Longevity data are fifth square-root transformed.

**Table 3:** Sire (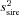) and dam (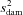) variance components and heritability estimates based on either of them for weight at emergence, developmental time, and longevity for separate sexes and all individuals. Estimates are presented with 95% confidence intervals (95% CI)

	*h* 2 (based on 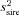 )	95% CI	*h*^2^ (based on 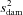 )	95% CI	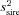	95% CI	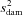	95% CI	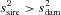
Weight at emergence^1^									
All	0.75	0.43; 1.03	0.35	0.24; 0.49	0.004	0.0028; 0.0075	0.002	0.0015; 0.0031	3
Males	1.12	0.74; 1.51	0.34	0.20; 0.51	0.009	0.0056; 0.0145	0.002	0.0017; 0.0041	3
Females	0.58	0.27; 0.90	0.60	0.37; 0.81	0.002	0.0011; 0.0039	0.002	0.0015; 0.0034	
Developmental time									
All	0.23	0.091; 0.38	0.31	0.20; 0.43	0.114	0.0522; 0.2083	0.152	0.0970; 0.2299	
Males	0.16	0.014; 0.33	0.33	0.14; 0.51	0.083	0.0134; 0.1862	0.167	0.0751; 0.2932	
Females	0.25	0.089; 0.44	0.34	0.18; 0.51	0.122	0.0489; 0.2339	0.167	0.0904; 0.2712	
Longevity^2^									
All	0.90	0.59	0.32	0.22; 0.57	0.006	0.0038; 0.0099	0.002	0.0014; 0.0032	3
Males	0.69	0.38	0.37	0.22; 0.57	0.004	0.0025; 0.0073	0.002	0.0013; 0.0037	
Females	1.43	0.96	0.22	0.10; 0.36	0.011	0.0069; 0.0176	0.001	0.0007; 0.0031	3

1 Weight at emergence data are log transformed.

2 Longevity data are fifth square-root transformed.

3 Significant difference between sire and dam variance component within each trait and sex.

**Table 4:** Genetic correlations for weight at emergence (W), developmental time (D), and longevity (L). (A) Cross sex genetic correlation (*r*_MF_) for each trait and (B) genetic correlation between traits (*r*_A_) for each sex. Presented as posterior mode and 95% HPD interval (95% HPD). Correlation is significantly different from zero/one when 95% CI is not including zero/one. All estimates are significantly different from one. Significant difference from zero is indicated in bold

(A)	*r*_MF_	95% HPD
Weight at emergence (W)	0.008	−0.258; 0.349
Developmental time (D)	**0.431**	0.0002; 0.630
Longevity (L)	0.168	−0.116; 0.442

(B)	Males		Females	
		
	*r*_A_	95% HPD	*r*_A_	95% HPD
W – D	0.032	–0.277; 0.347	0.005	–0.304; 0.295
L – W	−0.032	–0.290; 0.316	0.003	–0.305; 0.307
L – D	0.0005	–0.250; 0.357	0.047	–0.220; 0.392

## Discussion

In this study, we used a very common method (paternal half-sib design) to estimate the genetic architecture of life-history and morphological traits in the seed beetle *C. maculatus*. Our results highlight three important issues. First, we found major differences, as well as similarities, to other studies made on the same species and traits. This emphasizes the fact that estimates of quantitative genetic parameters are strictly population, time, and environment specific and results from one population should not be extrapolated to other populations, and certainly not other species. Second, we found real sex differences in heritability estimates and low genetic correlations between sexes (*r*_MF_) for life-history traits that are only moderately (weight at emergence) to slightly (longevity) sexually dimorphic. These interesting results highlight that sexes should not be assumed to be the same, and have great implications for the study of sexual dimorphism and its evolutionary dynamics. Third, we found sire variance components to be significantly higher than dam variance components for weight at emergence for males and for longevity for females, suggesting that nonadditive genetic and/or environmental effects might play a role in the estimation of heritability.

Heritability for weight at emergence and longevity was significant (Table 3), which is consistent with earlier studies (e.g., Møller et al. 1989; Fox 1993a; Tatar and Carey 1994), and heritability for developmental time was significant in both males and females (Table 3). These findings partly contradict previous results where significant heritability in this species was only detected in females (Fox 1994) or in none of the sexes (Fox et al. 2004). We found genetic correlations between the traits (*r*_A_) to be generally low and not significantly different from zero (Table 4B). Several studies have estimated *r*_A_ between these and similar traits, but different studies using the same experimental design report different results (Møller et al. 1989; Fox 1993a; Guntrip et al. 1997). For instance *r*_A_ for weight at emergence and developmental time was found to be significantly negative (Møller et al. 1989) or positive (Fox 1993a) but not different from zero (Guntrip et al. 1997). The low genetic correlations in our study suggests that different genes are influencing weight at emergence, developmental time, and longevity and that the response of each trait to selection is only partly (if at all) constraint by selection on correlated traits (i.e., those included in our study). Thus, an independent response of traits to selection is possible. Differences between studies are unlikely to be a result of the design per se, since we basically used the same design as used by others before (Fox 1993a; Messina 1993; Fox et al. 2004), but rather due to differences in host and population used. Our results highlight the fact that estimates of quantitative genetic parameters are strongly environment dependent and population specific (Kawecki 1995; Falconer and Mackay 1996; Simons and Roff 1996; Messina and Slade 1999; Messina and Fry 2003; Fox et al. 2004), and therefore should not be compared between studies, even for the same species.

### Sex-specific genetic effects

Importantly, we found sex-specific genetic effects for weight at emergence and adult longevity. Heritability was significantly higher in males compared to females for weight at emergence and significantly higher in females compared to males for longevity. The higher heritability is a result of the fact that the sire variance component in one sex (males for weight at emergence/females for longevity) is significantly higher than the sire variance component in the opposite sex (Table 2). Our results contradict earlier findings where heritability for body size and longevity in *C. maculatus* and *Stator limbatus* have been shown to rarely differ between males and females (Fox 1993a, 1994, 1998; Fox et al. 2003, 2004). Furthermore, we found the genetic correlation between sexes (*r*_MF_) for these traits to be low and not different from zero. Our findings are surprising and contradict results of previous studies, where genetic correlation between the sexes have been shown to be significantly different from zero and generally high (*r*_MF_ > 0.80) for both weight at emergence (Fox 1994) and adult life span (Fox et al. 2003).

*Callosobruchus maculatus* is known to be sexually dimorphic for the traits under observation (Møller et al. 1989; Guntrip et al. 1997) and our results confirm these findings. However, considering that these traits are only moderately (weight at emergence) to slightly (longevity) dimorphic, our findings regarding the underlying genetic architecture of traits and sexes are rather surprising. The extremely low genetic correlation between the sexes suggests that sexes share only a small part of the genome. Thus, sex-specific selection is likely to drive the sexes to their independent selective optima for the trait in question (Lande 1980, 1987; Reeve and Fairbairn 2001). The additive genetic variance determines the potential to respond to selection (Houle 1992). Our results suggest that the potential to respond to selection is substantially greater in one sex; males have a greater potential to respond to selection in their weight and females have a greater potential to respond in their longevity, and this response is not constraint by a high genetic correlation between the sexes. Thus, sexes are enabled to evolve independently. This could lead to a large degree of dimorphisms, given that different sexes experience different selection pressures on the traits in question due to different selective optima (Lande 1980, 1987; Reeve and Fairbairn 2001). Also, a recent study has confirmed that sex-specific genetic variances (apart from sex-specific environmental or maternal effects, Badyaev 2002) are an important contributor to the resolution of sexual genetic conflicts (Poissant et al. 2010, see also Bonduriansky and Rowe 2005; Fairbairn and Roff 2006; Fairbairn et al. 2007), leading to increased genetic independence between the sexes. Interestingly, our results suggest that an apparently great genetic independence might not correspond to a great phenotypic differences between the sexes.

There are various mechanisms that could potentially explain our findings. Different genes might be responsible for trait expression in males and females and/or genes have different influences in males and females and/or there is sex-specific gene expression of genes located on autosomes (Lande 1987; Rhen 2000), and/or sex-specific gene regulation. In addition, effects linked to sex chromosomes (Rice 1984; Reinhold 1998) and /or nonnuclear cytoplasmic genetic effects (e.g., mitochondria; Dowling et al. 2007) might play a role. We can exclude sex chromosome Y or X linkage as a general explanation to our findings, since the effect was found to be significant for either sex depending on the trait under observation (i.e., sire variance was larger for males compared to females for weight but smaller for males than females for longevity). However, there could still be trait-specific sex chromosome linkage, thus the sire effect could be explained to be due to Y linkage for weight at emergence but due to X linkage for longevity. If so, the sire effect on male offspring would cause a stronger resemblance than the sire effect on female offspring (100% compared to 50% extra resemblance due to male chromosome; Cowley et al. 1986; Cowley and Atchley 1988), meaning that we would expect the sire effect on weight at emergence to be stronger compared to the sire effect in longevity. However, the effect was found to be equally strong in weight at emergence and longevity, suggesting that a trait-specific Y- and X-linked effects are an unlikely explanation. Also, there is no evidence for cytoplasmic maternal effects in *C. maculatus* (Fox et al. 2004). Thus, a cyto–nuclear interaction does not in itself explain the sex differences in weight and longevity. Another possible cause of the elevated additive genetic variance is the presence of additive-by-additive epistasis (*V*_AA_), which is known to result in higher levels of additive genetic variance (Falconer and Mackay 1996; Lynch and Walsh 1998; Hallander and Waldmann 2007). This form of epistasis has also been shown to be present in morphological traits in the house fly (Meffert 2000). If so, there must be a component of sex linkage involved as these nonadditive gene actions affect male weight but female longevity. However, we estimated weight at emergence at three time points corresponding to early, median, and late emerging individuals. Since they are part of the same clutch (same genes interacting), but only with different emergence times, we would expect the nonadditive gene actions to be consistent over time. This was not the case in males, where the rank order changed over time, whereas the rank order was the same over time in females. This is not expected if the elevated sire variance is due to epistasis. Furthermore, since additive-by-additive epistasis contributes one-sixteenth to the sire component but one-fourth to the dam component (Falconer and Mackay 1996; Lynch and Walsh 1998), we would see an elevated dam component rather than an elevated sire component if epistasis is important. However, even if the epistatic interactions are sex limited to males, *V*_AA_ must be very large since *V*_A_ contributes four times more to the sire component than does *V*_AA_ (Falconer and Mackay 1996; Lynch and Walsh 1998).

### Sex-specific responses to environment

Our results could also be explained by sex differences that arise through sex-specific responses to the environment. The environment can influence males and females differently; one sex might be more vulnerable to a environmental condition than the other on both a genetic and phenotypic level. Sexes might for instance differ in how environmental factors affect and alter gene expression (Ellegren and Parsch 2007), the sex that is more genetically canalized shows lower additive genetic variance. But plasticity might also differ between sexes. For instance, Stillwell and Fox (2007) found a sex-specific plastic response to a change in the environment in *C. maculatus*. Males were generally more sensitive to rearing temperature than were females, creating variation in sexual size dimorphism. This implies that irrespective of how distinct or similar sexes are in their genetic makeup, the environment can lead to increased or decreased similarity of phenotypes, due to the fact that the genotypes of the two sexes differ in the slopes of the reaction norms across environments. This in itself could explain that even traits that have a potentially great genetic independence are only moderately or slightly dimorphic. Also, for the estimation of heritability the environment is important, since it is included in the estimate of heritability (phenotypic variation explained by the environment).

### Parental effects

Yet another factor that might play a role, are parental effects. Parental effects occur when the phenotype of an individual is affected by the phenotype or environment of its parents (Mousseau and Fox 1998). Many studies have shown that both paternal and especially maternal effects are present in this species (Fox 1993a; Messina 1998; Savalli et al. 2000; Fox et al. 2004). Interestingly, we found significant differences in sire and dam variance components within trait and sex for weight at emergence and longevity (Table 3). The sire variance component was significantly higher than the dam variance component for weight at emergence in males and for longevity in females. This difference in sire and dam variance components suggests the presence of nonadditive genetic and/or environmental effects (see Equation 1 and 2). This in turn could potentially explain the resulting sex differences in our *h*^2^ estimates. We suggest that this effect could possibly be explained by the biology of the species. *Callosobruchus maculatus* is a species without direct paternal or maternal care. However, males are known to provide females with nuptial jpgts during mating (in form of water and nutrition in the ejaculate; Boucher and Huignard 1987; Savalli and Fox 1999a, b; Edvardsson 2007; Ursprung et al. 2009). The ejaculate has been shown to have an overall positive effect on female fecundity (Fox 1993b; Savalli and Fox 1999a; Rönn et al. 2008) and adult life span (Fox 1993a). Thus, the observed effect could be due to a paternal effect on the weight at emergence of sons/longevity in daughters that is mediated via male ejaculate size, which in turn influences the condition of the female, and the size/longevity of her offspring. The influence of the condition of the mother on her offspring must be different for male compared to female offspring. However, this suggested scenario remains to be tested.

### Implications for the estimation of heritability

We found an increased sire compared to dam variance component within trait and sex. This increase was only detected in the traits and sexes where we also observed sex-specific variance components. As discussed, the difference in sire and dam variances suggests potential presence of environmental variance in the sire variance component. This in turn implies that an assumption of the breeding design is violated. In a paternal half-sib-full-sib design, the sire observational component is assumed to only include the additive genetic variance (barring epistasis), since maternal effects, dominance, and environmental effects (causal components) are controlled for with the breeding design and paternal effects are assumed to be absent. If now the sire variance component includes environmental variation (evidenced by 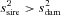), an overestimation of heritability for the trait under observation is the consequence. The widespread occurrence of both maternal and paternal nonadditive genetic and/or environmental effects represents a problem with all analyses of this kind. It must be stressed that our study was not in any way designed to estimate paternal effects (in contrary they are assumed to be absent), and thus this findings is highly unexpected. However, our findings suggests that studies where the sire variance component is lower than the dam variance component might not be as unbiased as assumed, since there might still be a nonadditive genetic and/or environmental effects inflating the estimate of the additive genetic variance. There is as yet no statistical model that would allow for the estimation of both maternal and paternal nonadditive genetic and/or environmental contributions from data of this type and our results make clear that the presence of nongenetic and/or indirect genetic environmental effects can largely inflate estimates, but to an unknown extent. This in turn suggests that all estimates of additive genetic variance, and heritability, using this, and similar methods should be treated with caution, as the assumption of sire variance components representing unbiased estimates of additive genetic variance might be violated. For studies in the field, complicated designs such as the North Carolina II (NCII) are most often not possible for biological or logistic reasons (Lynch and Walsh 1998). A study with the potential to draw conclusion about separate effects of parental genotype and common environment was conducted by Bilde et al. (2008). By applying a full diallel design with reciprocal crosses (using inbred lines of *C. maculatus*), they were able to estimate separate variance components for maternal and paternal genotype and common environment effects. Interestingly, Bilde et al. (2008) only found paternal effects to be significant for all of the traits measured (lifetime egg production, lifetime offspring production, and egg-to-adult survival). These results highlight the importance of paternal effects in general and show a step in the direction of an explicit separation of maternal and paternal effect and the possibility to draw conclusions about separate effects of maternal/paternal genotype and common environment. Overall, our finding emphasizes that the role of indirect effects (genetic or environmental) might be seriously underestimated and needs far more attention in future studies (e.g., Moore et al. 1997; Bijma and Wade 2008; McGlothlin et al. 2010).

### Main conclusion

We found real sex differences in additive genetic variance for life-history and morphological traits that are only moderate (weight at emergence) to slightly (longevity) sexually dimorphic. This highlights that sexes should not be treated the same when it comes to estimates of quantitative genetic parameters. Many quantitative genetic studies have focused on one sex only (primarily females) or simply ignored sexes. However, if sex differences are present, then estimates cannot be extrapolated to the population as a whole. Sex-specific genetic variance and *r*_MF_ play an important role for sexual dimorphism, its evolutionary dynamics, and (the resolution of) sexual genetic conflicts (Bonduriansky and Rowe 2005; Fairbairn and Roff 2006; Fairbairn et al. 2007; Poissant et al. 2010). Moreover, they might even have implications for population dynamics and even speciation, since for instance sexual dimorphism has been shown to correlate with population fitness (Kokko and Brooks 2003; Rankin and Arnqvist 2008). Thus, future quantitative genetic studies would certainly benefit from incorporating sex as yet another dimension in multivariate genetic analysis.
